# Psychometric properties of the arabic translation of the Physical Appearance Comparison Scale-Revised (PACS-R) in adults

**DOI:** 10.1186/s40359-024-01871-x

**Published:** 2024-06-29

**Authors:** Marie Anne El Khoury, Diana Malaeb, Mirna Fawaz, Nancy Chammas, Michel Soufia, Feten Fekih-Romdhane, Sahar Obeid, Souheil Hallit

**Affiliations:** 1 Social and Education Sciences Department, School of Arts and Sciences, Lebanese American University, Beirut, Lebanon; 2 College of Pharmacy, Gulf Medical University, Ajman, United Arab Emirates; 3 College of Health Sciences, American University of the Middle East, Kuwait, Kuwait; 4 School of Medicine and Medical Sciences, Holy Spirit University of Kaslik, P.O. Box 446, Jounieh, Lebanon; 5 The Tunisian Center of Early Intervention in Psychosis, Department of Psychiatry “Ibn Omrane”, Razi hospital, Manouba, 2010 Tunisia; 6 Faculty of Medicine of Tunis, Tunis El Manar University, Tunis, Tunisia; 7 Psychology Department, College of Humanities, Effat University, Jeddah, 21478 Saudi Arabia; 8 Applied Science Research Center, Applied Science Private University, Amman, Jordan

**Keywords:** Physical appearance, Appearance comparison, Body image, Psychometrics properties, Arabic validation

## Abstract

**Background:**

Physical comparison may be a factor in body dissatisfaction and related issues, like eating disorders and depression. The Physical Appearance Comparison Scale-Revised (PACS-R) is a scale developed to assess the frequency of physical comparison. Because there is no validated scale for body comparison in Arabic, this study aims to address this gap by validating the PACS-R in the Arabic language.

**Methods:**

The PACS-R was translated to Arabic following a conventional forward-backward translation procedure, and was administered to a sample of 359 Lebanese adults along with The Depression Anxiety Stress Scale, and the Rosenberg self-esteem scale (RSES) for convergent validity. The factor structure was studied by confirmatory factor analysis (CFA), and composite reliability was assessed using McDonald’s omega and Cronbach’s alpha.

**Results:**

Results suggested a one-factor structure of the Arabic PACS-R, with good internal consistency (McDonald’s ω = 0.97 / Cronbach α = 0.97). Measurement invariance was established across sex groups, with no significant difference being reported between males and females in terms of PACS-R scores (15.42 ± 10.64 vs. 13.16 ± 11.88; t(357) = 1.84; *p* = .066). Finally, adequate convergent validity was tested and found to be adequate, with PACS-R scores found to be correlated negatively with self-esteem and positively with psychological distress.

**Conclusion:**

The present findings preliminarily establish the Arabic PACS-R as an effective instrument for researchers and practitioners aiming to explore the physical comparison among Arabic-speaking populations, thus contributing to research and clinical work in the Arabic community.

**Supplementary Information:**

The online version contains supplementary material available at 10.1186/s40359-024-01871-x.

## Introduction

Body dissatisfaction represents a pervasive concern within contemporary society, impacting individuals across various age groups, genders, and cultural backgrounds [1, 2]. Furthermore, it is a core symptom of eating disorders [3] and one of its leading causes [4]. Besides, it is also involved in depression and low self-esteem and this was found to affect both sexes, teenagers and adults [1, 5–10]. This is a multifaceted phenomenon influenced by psychological, sociocultural, and environmental factors [2, 11, 12]. It can stem from comparison with societal ideals, often internalized through media exposure and reinforced by peer and familial attitudes. This leads us to the theory of social comparison, first introduced by Festinger in 1954 [13], where he suggests that people have a natural drive to evaluate their own opinions and abilities. When lacking objective measures, they instinctively compare themselves to others. The comparison can be upward or downward: when individuals can compare themselves with others perceived to be superior or inferior in some way. This theory can be applied to different psychological and social contexts, notably body image [12, 14, 15]. In the context of body image, this theory has played a key role in understanding how comparative evaluation with peers, media portrayals, and societal beauty norms shape individual perceptions of their physical attractiveness and value [16, 17]. It has been recognized that unintended comparisons can take place, and the benchmark used in the comparison might involve someone quite different from oneself [12].

Research highlights the potentially harmful effects of engaging in social comparisons based on appearance, whether it is peer comparison or social media comparison [14, 17–19]. Based on the social comparison theory, comparison can happen upward toward idealized body images portrayed by social media and television which frequently results in feelings of insufficiency, dissatisfaction with one’s body, and a negative self-image [19, 20]. Thus, upward comparisons are linked to a more negative impact than downward comparisons [21, 22]. Moreover, comparison with media tends to have a more harmful effect [21, 23]. Social comparison and more specifically appearance comparison are associated with body dissatisfaction, disordered eating, and low self-esteem [14, 24]. Social comparison correlates positively with psychological distress [25], depression and anxiety [26]. Furthermore, physical comparison was seen to be associated with higher anxiety [24, 27] and depression [28]. Furthermore, sex differences appear to exist in physical social comparison, leading to differential negative effects on males compared to females. Females seem to be more inclined to compare their appearances to others than men, which is associated with several negative psychological outcomes such as lower self-esteem, depression, body dissatisfaction, and dieting behaviors [17, 29]. While males also engage in appearance comparisons, they do so less frequently and with fewer negative consequences for their body image [30]. Overall, this body of research underscores the significant, and often harmful, impact of appearance comparisons on both females’ and males’ mental health and body image, with a stronger effect observed in females [31]. Considering the significant role that appearance comparisons play in issues related to body image and eating disorders, it is crucial to possess a tool that effectively measures an individual’s propensity for engaging in physical appearance comparisons.

### Measurement instruments of physical appearance comparison

Different scales have been created to evaluate the inclination towards appearance comparison, but most come with considerable drawbacks. Among the first to be developed is the Body Comparison Scale (BCS; [11], evaluating the frequency with which an individual compares specific parts of their body with others. However, a notable limitation of this tool is its failure to directly compare one’s weight or body fat [31]. Additionally, the scale lacks details about the comparison’s target and the context, both of which are vital for understanding the dynamics and potential triggers of appearance comparisons. O’Brien et al. [32] introduced scales designed to measure the propensity for engaging in comparisons with those deemed significantly more attractive (Upward Physical Appearance Comparison Scale, UPACS) and those considered much less attractive (Downward Appearance Comparison Scale, DACS). Their validation was confined to the Chinese cultural milieu, wherein their psychometric characteristics were found to be satisfactory [33]. Nonetheless, Schaefer and Thompson [31] raised critiques regarding the UPACS and DACS scales, pointing out that these scales judge appearance comparisons through the lens of attractiveness stereotypes and do not cover lateral comparisons, where individuals compare themselves to others of perceived similar attractiveness, consequently, they might only offer a narrow view of the frequency with which individuals engage in appearance comparisons.

The physical appearance scale (PACS), created by Thompson et al. in 1991 [34], was considered one of the primary validated tools for assessing how individuals compare their looks with others [17]. It is a 5-item scale primarily developed for females, thus sex differences in body image concerns highlight a potential limitation of the original PACS, as males and females aspire to different physical ideals, which may not be fully captured by the scale. The Physical Appearance Comparison Scale-Revised (PACS-R) addressed this issue, among others, including the evaluation of weight and shape and a wider variety of comparison contexts [31]. The PACS-R demonstrates great internal consistency (Cronbach’s alpha of 0.97), featuring 11 items phrased neutrally and encompassing a broader range of contexts for evaluation. Exploratory factor analysis and parallel analysis suggested a single-factor structure for the PACS-R, as well as strong convergent validity with indices of body satisfaction, eating disorders, the impact of sociocultural standards on appearance, and self-esteem among female college students [31]. The PACS-R has been translated and validated in different languages among which are Spanish [35], Iranian [36], and Brazilian Portuguese [37] all if which found single-factor structures. On another hand, high physical comparison associates with low self-esteem as seen in the original study of PACS-R [31] and other validation studies [35, 37]. Strong associations were found with eating disorders and stress [31, 35, 37], aligning with previous research that shows association of physical comparison with stress, anxiety and depression [38, 39]. To date, however, there has been no translation and validation of the PACS-R into the Arabic language.

### The present study

In the Arab world, around one-third of females display restrictive eating patterns [40]. Several studies [41–48] showed how media exposure, societal and peer pressures, and individual factors (like sex, age, and BMI) contribute to body image concerns and eating disorders in the Arab context. The findings highlight a need for comprehensive health education, media literacy initiatives, and mental health support tailored to the unique cultural and societal framework of the Arab world. These efforts aim to mitigate the impact of negative body image and eating disorders among youth, advocating for a healthier, more inclusive understanding of body image and self-esteem. Along these lines, an Arabic version of the PACS-R is needed to address the physical comparison in Arabic-speaking populations. Moreover, applying the social theory to the Arab world, findings show that higher levels of collectivism are linked with a greater overall inclination to engage in comparison, a heightened interest in making upward comparisons, and a reduced interest in making downward comparisons [49]. Hofstede [50] posits that Arab nations are characterized by a collectivist cultural orientation, thus social comparison has a considerable impact. While the concept of body comparison holds significant importance, the absence of a validated Arabic measure stands as a gap. Advancing research in this field necessitates the creation of reliable and valid tools. The current study has the following objectives: first, to analyze the factor structure and assess the model fit of the PACS-R adapted into Arabic; second, to investigate the consistency of their measurement across sex; and third, to evaluate the validity of our Arabic translated version of the PACS-R by exploring its association with self-esteem and psychological distress. Our hypothesis suggests that the Arabic PACS-R would reveal a unidimensional structure with a satisfactory level of internal consistency and would display measurement invariance across sex. Moreover, we anticipate that the PACS-R would have positive correlation with psychological distress and negative correlation with self-esteem.

## Methods

### Study design and participants

A total of 359 Lebanese participants were enrolled in this cross-sectional study that was conducted between September and November 2022, through convenience sampling in several Lebanese governorates. The research team approached people and asked them to fill the survey; those who accepted were asked to forward the link to other people they might know, explaining the snowball sampling technique followed. The survey was a Google form questionnaire that was administered through the internet, using the snowball technique. Participants were informed about the study, and were provided an online link to it; pressing on the link led interested participants to the consent form and information form (outlining the current study’s objectives, anonymity, and voluntary permission to research). When confidentiality is assured, participants are encouraged to respond honestly and deliver more accurate information. Secondly, detailed instructions defining the purpose of the survey and the importance of the thoughtfulness of the responses minimized inaccuracy. No rewards were given to participants in return for participation.

### Measures

The questionnaire used was anonymous and in Arabic, the native language in Lebanon. It required approximately 10 to 15 min to complete. It consisted of three parts. The first part explained the study’s topic and objective, a statement ensuring the anonymity of respondents. The participant had to select the option stating “I consent to participate in this study” to be directed to the questionnaire.

#### Sociodemographic survey

Participants provided self-reports on their age, sex, marital status, body mass index (calculated from self-reported weight and height) and the household crowding index, which reflects the socioeconomic status (calculated by dividing the number of persons by that of the rooms in the house besides the kitchen and bathrooms) [51].

**Revised Physical Appearance Comparison** Scale (PACS-R [31]: The PACS-R is comprised of an 11-item survey designed to assess how often individuals compare their physical appearance to that of others across a wide range of social contexts. Responses are collected using a 5-point Likert scale, with options extending from “Never” to “Always.” A higher score on the scale signifies a greater frequency of appearance comparison. The Arabic version of the PACS-R scale was translated and culturally adapted before being used in this study. This involved translating the scale into Arabic in line with international standards and recommendations to ensure semantic equivalence between the original measurements and their Arabic counterparts [52]. We used forward and back-translation procedure. The Arabic version was initially translated from English by a Lebanese translator. Subsequently, a Lebanese psychologist fluent in English retranslated the Arabic text back into English, ensuring that each translation, whether specific or literal, was suitable. In addition to the study team, two psychiatrists and a psychologist reviewed both the original and retranslated English version to identify and rectify any discrepancies, ensuring the accuracy of the translation. A specialized measure was implemented to confirm that the Arabic and the original versions are conceptually equivalent. This step was designed to address any potential misunderstandings concerning the language and readability of the items [53]. A pilot study was conducted on 20 persons before the start of the official data collection to make sure all questions are well understood; no changes were done consequently.

#### The DASS scale

The Depression Anxiety Stress Scales [54] is a self-report questionnaire created to quantify three negative emotional states: depression, anxiety, and stress. We used a shorter version of 8 items (DASS-8, [55] that has demonstrated high validity and reliability. It is composed of three subscales with: depression (3 items, ω = 0.82 / α = 0.82), anxiety (3 items, ω = 0.81 / α = 0.81) and stress (2 items, α = 0.68). Items are rated on a four-point scale from 0 to 3.

#### The Rosenberg Self-Esteem scale (RSES)

The RSES [56] was employed to assess trait self-esteem. This instrument includes 10 items, half of which are reverse-scored. It utilizes a 4-point Likert scale ranging from “Strongly Disagree” to “Strongly Agree,” where higher scores signify greater self-esteem. The scale has been previously utilized in its Arabic-translated form in various studies [57, 58].

### Analytic Strategy

#### Confirmatory factor analysis

There were no missing responses in the dataset. We used data from the total sample to conduct a CFA using the SPSS AMOS v.26 software. We aimed to enroll a minimum of 220 adolescents following the recommendations of Mundfrom et al. of 3 to 20 times the number of the scale’s variables [59]. Parameter estimates were obtained using the maximum likelihood method. Multiple fit indices were calculated: Steiger-Lind root mean square error of approximation (RMSEA), standardized root mean square residual (SRMR), Tucker-Lewis Index (TLI), and Comparative Fit Index (CFI). Values ≤ 0.08 for RMSEA, ≤ 0.05 for SRMR and ≥ 0.90 for CFI and TLI indicate a good fit of the model to the data [60]. Additionally, values of the average variance extracted (AVE) ≥ 0.50 indicated evidence of convergent validity [61]. Multivariate normality was not verified at first (Bollen-Stine bootstrap *p* = .002); therefore we performed a non-parametric bootstrapping procedure.

#### Sex invariance

To examine gender invariance of PACS-R scores, we conducted multi-group CFA [62] using the total sample. Measurement invariance was assessed at the configural, metric, and scalar levels [63]. We accepted ΔCFI ≤ 0.010 and ΔRMSEA ≤ 0.015 or ΔSRMR ≤ 0.010 as evidence of invariance [64].

#### Further analyses

Composite reliability was assessed using McDonald’s ω and Cronbach’s α, with values greater than 0.70 reflecting adequate composite reliability [65]. Normality was verified since the skewness and kurtosis values for each item of the scale varied between − 1 and + 1 [66]. Pearson test was used to correlate the PACS-R scores with the other scales in the survey. Student t test was used to compare two means.

## Results

### Participants

Three hundred fifty-nine participants participated in this study, with a mean age of 22.75 ± 7.04 years (age range 18–58), 59.9% females and 92.2% single. In addition, the mean BMI was 24.12 ± 512 kg/m^2^ and the mean HCI was 1.28 ± 1.92 person/room.

### Confirmatory factor analysis of the PACS-R scale

**Fig. 1:**
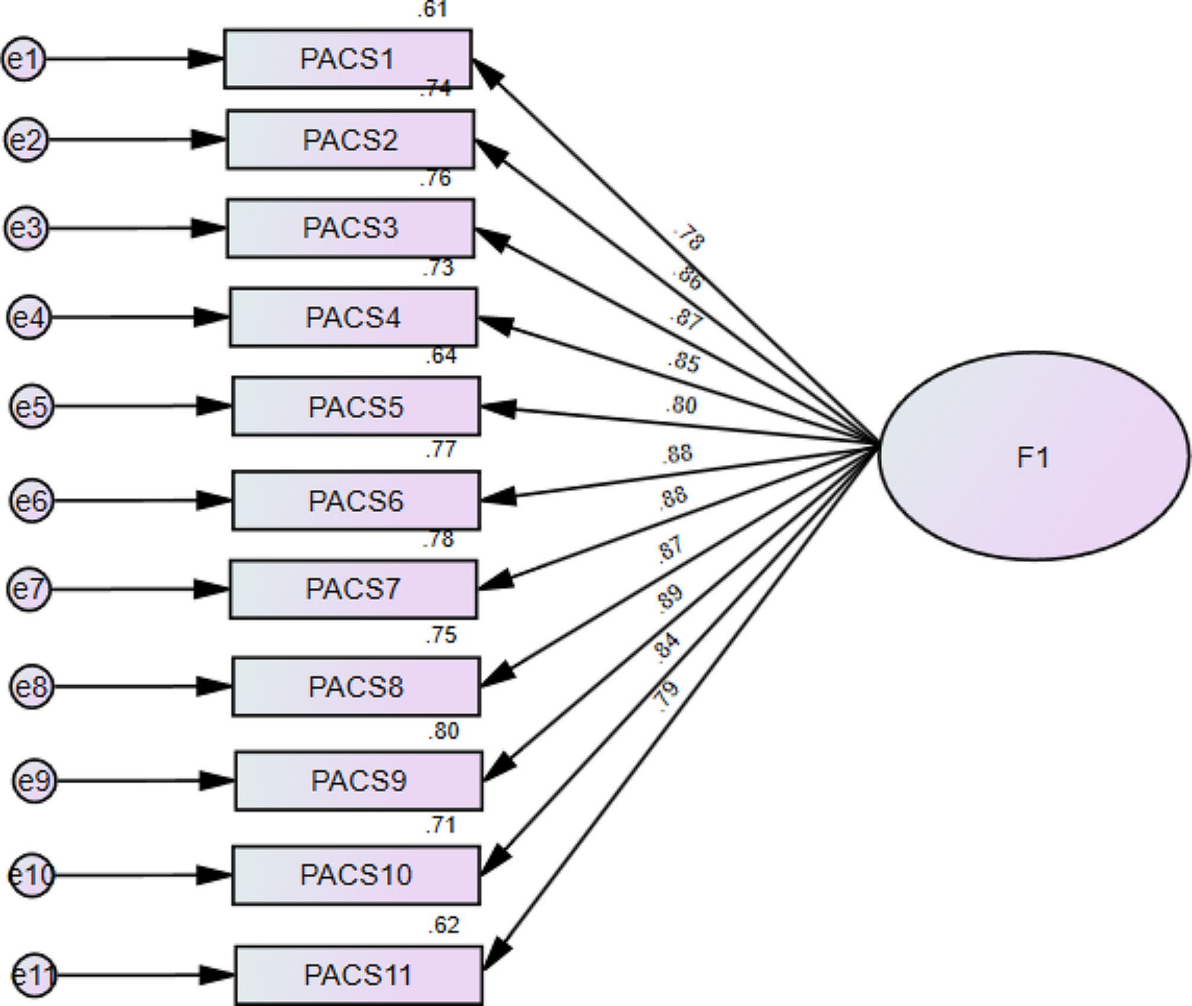
Standardized loading factors of the Physical Appearance Comparison Scale-Revised (PACS-R) items in Arabic

CFA indicated that fit of the one-factor model of the PACS-R scale was acceptable: RMSEA = 0.125 (90% CI 0.112, 0.139), SRMR = 0.031, CFI = 0.940, TLI = 0.924. The standardized estimates of factor loadings were all adequate (Fig. 1). Composite reliability of scores was adequate in the total sample (ω = 0.97 / α = 0.97). The convergent validity for this model was very good, as AVE = 0.72.

### Gender invariance

We were able to show the invariance across sex at the configural, metric, and scalar levels (Table 1). No significant difference was seen between males and females in terms of PACS-R scores (15.42 ± 10.64 vs. 13.16 ± 11.88; *t*(357) = 1.84; *p* = .066).

**Table 1:** Measurement invariance of the physical appearance comparison scale across gender in the total sample

Model	CFI	RMSEA	SRMR	Model Comparison	ΔCFI	ΔRMSEA	ΔSRMR
Males	0.907	0.146	0.049				
Females	0.920	0.155	0.033				
Configural	0.915	0.107	0.049				
Metric	0.915	0.102	0.053	Configural vs. metric	< 0.001	0.005	0.004
Scalar	0.912	0.098	0.052	Metric vs. scalar	0.003	0.004	0.001

*Note* CFI = Comparative fit index; RMSEA = Steiger-Lind root mean square error of approximation; SRMR = Standardised root mean square residual

### Concurrent validity

Higher physical appearance comparison scores were significantly associated with lower self-esteem (*r* = − .43; *p* < .001) and higher psychological distress (*r* = .37; *p* < .001).

## Discussion

The objective of this study was to translate the PACS-R into Arabic and to examine its psychometric properties in terms of factor structure, internal consistency reliability, cross-sex measurement invariance and concurrent validity. To this end, CFA, reliability evaluation, and correlational analysis were conducted. The findings in our study support the satisfactory psychometric characteristics of the Arabic iteration of the PACS-R. The evaluation of the Arabic PACS-R in a sample of Arabic-speaking Lebanese adults identified a single-factor structure with all 11 items retained, which aligns with the original model [31]. As expected, the Arabic PACS-R also exhibited good reliability and concurrent validity, suggesting its suitability for use among Arabic-speaking adults in community settings.

Our results share the single-factor structure with the original scale validation [31], where they initially considered a multi-factor solution but ultimately supported a single-factor solution through additional analyses. Similar findings supporting the one-dimensional structure were observed in the subsequent translation validations [35–37]. Our findings also showed that composite reliability of the Arabic version of the PACS-R was excellent (ω = 0.97 / α = 0.97). These high values indicate that the scale items are both consistent and effectively measure the same underlying construct. This is supported across the validations in different languages, where high internal consistency, with Cronbach’s alpha and McDonald’s omega values consistently above 0.95, indicating excellent reliability [35–37].

Sex invariance of the Arabic PACS-R was established, indicating the scale’s applicability across sexes. This is aligned with another mixed sex PACS-R validation [35]. This means the PACS-R scale measures the same construct in the same way for both males and females, allowing for direct comparisons. Since other validations used a female-only sample only [36, 37], this study is one of the few along with the Spanish version that validated the scale on a mixed sex sample. Our sample had a good ratio of males and females which showcases a significant strength of the Arabic PACS-R version. As for between-sex comparisons, our study showed no significant difference across sex in terms of PACS-R scores. This comes in contrast with the findings from the Spanish translation, where a significant sex difference was observed in PACS-R scores, highlighting the influence of sex on physical appearance comparison concerns [35]. These differences can be attributed to sample demographics, the characteristics of the study samples (such as age range, social and economic backgrounds), and the specific population sampled (e.g., university students, general population). For example, a sample composed of a majority of young adults from a university setting might reflect more homogeneous attitudes toward appearance, potentially minimizing or exaggerating sex differences seen in a broader, more diverse population.

Finally, the Arabic PACS-R showed good patterns of convergent validity with measures of self-esteem and psychological distress. In particular, increased frequency of body comparison correlated with low self-esteem. This is supported by the original validation study of the PACS-R [31], as well as other studies [24, 35, 37]. Correspondingly, greater PACS-R scores also correlated with increased psychological distress. These outcomes align with the conclusions of previous research that link increased physical comparison with higher level of depression and anxiety [67, 68]. Indeed, low self-esteem has been linked to upward social comparison [18]. This relation seems bidirectional, as upward social comparison appears to lower self-esteem, but also people with low self-esteem and negative mood tend to engage in upward social comparisons [19, 20]. Additionally, physical appearance is recognized as one of the most prominent aspects of self-esteem, especially among teenagers and young adults [69]. Thus, having an association between higher PACS-R and lower self-esteem and psychological distress might fall under this bidirectional relation. Moreover, upward social comparisons have also been associated with additional adverse outcomes, such as depressive symptoms [26, 70, 71] and self-esteem has been demonstrated to partially mediate the relationship between depressive symptoms and upward social comparisons [69, 72].

### Study limitations

The current study’s limitations should be acknowledged. Primarily, the data were collected through convenience (non-probabilistic) and web-based sampling methods, which might restrict the extrapolation of our findings. The sample was mostly comprised of Lebanese young adults, with slightly more females than males, which may limit the applicability of the findings to broader demographic populations. Furthermore, we need to take into account cultural differences between other Arabic-speaking countries that may differ from our Lebanese sample. The Arabic PACS-R needs further validation across different demographics, including older participants and from different Arabic-speaking countries. Next, the reliance on self-reported surveys may introduce the potential biases related to memory recall and social desirability. Finally, certain critical psychometric properties of the PACS-R, such as test-retest reliability have not been assessed. These aspects warrant further examination in subsequent research.

## Conclusion

Despite these limitations, the study offers substantial evidence that the Arabic version of the PACS-R possesses robust psychometric qualities. The comprehensive results preliminarily establish the Arabic PACS-R as an effective instrument for researchers and practitioners aiming to explore the physical comparison among Arabic-speaking populations, thus contributing to research and clinical work in the Arabic community. Future studies across the lifespan (e.g., adolescents) using larger populations of Arabic-speaking adults from different countries, as well as clinical samples are required to confirm the present findings.

### Electronic supplementary material

Below is the link to the electronic supplementary material.

Supplementary Material 1

## Data Availability

The datasets generated and/or analysed during the current study are not publicly available due to restrictions from the ethics committee but are available from the corresponding author on reasonable request.

## References

[1] Gogolin T, Norris E, Murch H, Volk F. The effect of male body dissatisfaction on sexual and relationship satisfaction in the Presence of Pornography Use and Depression. J Men’s Stud. 2024;10608265241236478. 10.1177/10608265241236478.

[2] Holmqvist K, Frisén A (2010). Body dissatisfaction across cultures: findings and research problems. Eur Eat Disorders Rev.

[3] American Psychiatric Association. Diagnostic and statistical manual of mental disorders: DSM-5. 5th ed. 2013.

[4] Stice E, Shaw HE (2002). Role of body dissatisfaction in the onset and maintenance of eating pathology: a synthesis of research findings. J Psychosom Res.

[5] Barnes M, Abhyankar P, Dimova E, Best C (2020). Associations between body dissatisfaction and self-reported anxiety and depression in otherwise healthy men: a systematic review and meta-analysis. PLoS ONE.

[6] Brechan I, Kvalem IL (2015). Relationship between body dissatisfaction and disordered eating: mediating role of self-esteem and depression. Eat Behav.

[7] Chen J, Peng S, Wei Y (2024). New media facilitate adolescents’ body dissatisfaction and eating disorders in Mainland China. Trends Mol Med.

[8] Corno G, Paquette A, Burychka D, Miragall M, Rivard M-C, Baños RM, Bouchard S (2024). Development of a visual-perceptual method to assess body image: a cross-cultural validation in Canadian and Spanish women. Eur Eat Disorders Review: J Eat Disorders Association.

[9] Faria K, Haçul BE, Lopes J, de Andrade GF, SEXUAL DISSATISFACTION OF WOMEN ASSISTED IN A BASIC HEALTH UNIT (2024).

[10] Karazsia BT, Murnen SK, Tylka TL (2017). Is body dissatisfaction changing across time? A cross-temporal meta-analysis. Psychol Bull.

[11] Fisher E, Dunn M, Thompson JK (2002). Social comparison and body image: an investigation of body comparison processes using Multidimensional Scaling. J Soc Clin Psychol.

[12] Morrison TG, Kalin R, Morrison MA (2004). Body-image evaluation and body-image investment among adolescents: a test of sociocultural and social comparison theories. Adolescence.

[13] Festinger L (1954). A theory of social comparison processes. Hum Relat.

[14] Bailey SD, Ricciardelli LA (2010). Social comparisons, appearance related comments, contingent self-esteem and their relationships with body dissatisfaction and eating disturbance among women. Eat Behav.

[15] Krayer A, Ingledew DK, Iphofen R (2008). Social comparison and body image in adolescence: a grounded theory approach. Health Educ Res.

[16] Fitzsimmons-Craft EE, Harney MB, Koehler LG, Danzi LE, Riddell MK, Bardone-Cone AM (2012). Explaining the relation between thin ideal internalization and body dissatisfaction among college women: the roles of social comparison and body surveillance. Body Image.

[17] Myers TA, Crowther JH (2009). Social comparison as a predictor of body dissatisfaction: a meta-analytic review. J Abnorm Psychol.

[18] Vogel E, Rose J, Roberts L, Eckles K (2014). Social Comparison, Social Media, and self-esteem. Psychol Popular Media Cult.

[19] Vogel EA, Rose JP, Okdie BM, Eckles K, Franz B (2015). Who compares and despairs? The effect of social comparison orientation on social media use and its outcomes. Pers Indiv Differ.

[20] Tibber MS, Zhao J, Butler S (2020). The association between self-esteem and dimensions and classes of cross-platform social media use in a sample of emerging adults – evidence from regression and latent class analyses. Comput Hum Behav.

[21] Schaefer L, Thompson J. The Development and Validation of the physical appearance comparison Scale-3 (PACS-3). Psychol Assess. 2018;30. 10.1037/pas0000576. 10.1037/pas0000576 PMC6942695 29781660

[22] Thompson JK, Heinberg LJ, Altabe M, Tantleff-Dunn S. Exacting beauty: theory, assessment, and treatment of body image disturbance. American Psychological Association; 1999.

[23] Ridolfi D, Myers T, Crowther J, Ciesla J (2011). Do Appearance focused cognitive distortions moderate the relationship between Social Comparisons to peers and media images and body image disturbance?. Sex Roles.

[24] Alcaraz-Ibáñez M, Sicilia Á, Díez-Fernández DM, Paterna A (2020). Physical appearance comparisons and symptoms of disordered eating: the mediating role of social physique anxiety in Spanish adolescents. Body Image.

[25] Webb caroline. (2000). *Psychological distress in clinical obesity: The role of eating disorder beliefs and behaviours, social comparison and shame*. ProQuest Dissertations & Theses Global. https://www.proquest.com/dissertations-theses/psychological-distress-clinical-obesity-role/docview/900298535/se-2.

[26] McCarthy PA, Morina N (2020). Exploring the association of social comparison with depression and anxiety: a systematic review and meta-analysis. Clinical Psychology Psychotherapy.

[27] Rapee RM, Magson NR, Forbes MK, Richardson CE, Johnco CJ, Oar EL, Fardouly J (2022). Risk for social anxiety in early adolescence: longitudinal impact of pubertal development, appearance comparisons, and peer connections. Behav Res Ther.

[28] Schlechter P, Morina N (2023). The role of aversive appearance-related comparisons and self-discrepancy in Depression and Well-being from a Longitudinal General Comparative-Processing Perspective. Behav Ther.

[29] Keery H, van den Berg P, Thompson JK (2004). An evaluation of the tripartite influence model of body dissatisfaction and eating disturbance with adolescent girls. Body Image.

[30] Karazsia BT, Crowther JH (2009). Social body comparison and internalization: mediators of social influences on men’s muscularity-oriented body dissatisfaction. Body Image.

[31] Schaefer LM, Thompson JK (2014). The development and validation of the physical appearance comparison scale-revised (PACS-R). Eat Behav.

[32] O’Brien KS, Caputi P, Minto R, Peoples G, Hooper C, Kell S, Sawley E (2009). Upward and downward physical appearance comparisons: development of scales and examination of predictive qualities. Body Image.

[33] Liao J, Jackson T, Chen H (2014). The structure and validity of directional measures of appearance social comparison among emerging adults in China. Body Image.

[34] Thompson J, Heinberg L, Tantleff-Dunn S (1991). The physical appearance comparison scale. Behav Therapist.

[35] Vall Roqué H, Andrés A, Saldaña C (2022). Validation of the Spanish version of the physical appearance comparison scale-revised (PACS-R): psychometric properties in a mixed-gender community sample. Behav Psychology/Psicologia Conductual.

[36] Atari M, Akbari-Zardkhaneh S, Soufiabadi M, Mohammadi L. (2015). Cross-Cultural Adaptation of the Physical Appearance Comparison Scale-Revised in Iran. *International Journal of Body, Mind and Culture*, *2*.

[37] Claumann GS, Laus MF, Folle A, Silva DAS, Pelegrini A (2021). Translation and validation of the Brazilian version of the physical appearance comparison scale-revised in college women. Body Image.

[38] Cattarin JA, Thompson JK, Thomas C, Williams R (2000). Body image, Mood, and televised images of attractiveness: the role of Social Comparison. J Soc Clin Psychol.

[39] McCreary DR, Saucier DM (2009). Drive for muscularity, body comparison, and social physique anxiety in men and women. Body Image.

[40] Fekih-Romdhane F, Hallit R, Malaeb D, Sakr F, Dabbous M, Sawma T, Obeid S, Hallit S (2023). Psychometric properties of an arabic translation of the Nine Item Avoidant/Restrictive Food Intake Disorder Screen (NIAS) in a community sample of adults. J Eat Disorders.

[41] Alharballeh S, Dodeen H (2023). Prevalence of body image dissatisfaction among youth in the United Arab Emirates: gender, age, and body mass index differences. Curr Psychol.

[42] Al-Musharaf S, Rogoza R, Mhanna M, Soufia M, Obeid S, Hallit S (2022). Factors of body dissatisfaction among Lebanese adolescents: the indirect effect of self-esteem between mental health and body dissatisfaction. BMC Pediatr.

[43] Haddad C, Zakhour M, Akel M, Honein K, Akiki M, Hallit S, Obeid S (2019). Factors associated with body dissatisfaction among the Lebanese population. *Eating and Weight disorders*-*studies on Anorexia*. Bulimia Obes.

[44] Hallit S, Mhanna M, Soufia M, Obeid S (2022). Factors of body dissatisfaction among Lebanese adolescents: the indirect effect of self-esteem between mental health and body dissatisfaction. BMC Pediatr.

[45] Mousa TY, Mashal RH, Al-Domi HA, Jibril MA (2010). Body image dissatisfaction among adolescent schoolgirls in Jordan. Body Image.

[46] Musaiger AO, Al-Mannai M, ASSOCIATION BETWEEN EXPOSURE TO MEDIA AND BODY WEIGHT CONCERN AMONG FEMALE UNIVERSITY STUDENTS IN FIVE ARAB COUNTRIES (2014). A PRELIMINARY CROSS-CULTURAL STUDY. J Biosoc Sci.

[47] Obeid S, Chok A, Sacre H, Haddad C, Tahan F, Ghanem L, Azar J, Hallit S (2021). Are eating disorders associated with bipolar disorder type I? Results of a Lebanese case-control study. Perspect Psychiatr Care.

[48] Schulte SJ, Thomas J (2013). Relationship between eating pathology, body dissatisfaction and depressive symptoms among male and female adolescents in the United Arab Emirates. Eat Behav.

[49] Chung T, Mallery P (1999). Social comparison, individualism-collectivism, and self-esteem in China and the United States. Curr Psychol.

[50] Hofstede G. Dimensionalizing cultures: the Hofstede Model in Context. Online Readings Psychol Cult. 2011;2(1). 10.9707/2307-0919.1014.

[51] Melki IS, Beydoun HA, Khogali M, Tamim H, Yunis KA, National Collaborative Perinatal Neonatal Network (NCPNN) (2004). Household crowding index: a correlate of socioeconomic status and inter-pregnancy spacing in an urban setting. J Epidemiol Commun Health.

[52] van Widenfelt BM, Treffers PDA, de Beurs E, Siebelink BM, Koudijs E (2005). Translation and cross-cultural adaptation of assessment instruments used in psychological research with children and families. Clin Child Fam Psychol Rev.

[53] Ambuehl B, Inauen J (2022). Contextualized measurement scale adaptation: a 4-Step tutorial for health psychology research. Int J Environ Res Public Health.

[54] Brown TA, Chorpita BF, Korotitsch W, Barlow DH (1997). Psychometric properties of the Depression anxiety stress scales (DASS) in clinical samples. Behav Res Ther.

[55] Ali AM, Hori H, Kim Y, Kunugi H (2022). The Depression anxiety stress scale 8-Items expresses Robust Psychometric properties as an Ideal Shorter Version of the Depression anxiety stress scale 21 among healthy respondents from three continents. Front Psychol.

[56] Rosenberg M. (1965). *Rosenberg self-esteem scale (RSE). Acceptance and commitment therapy Measures package*.

[57] Obeid S, Haddad C, Zakhour M, Fares K, Akel M, Salameh P, Hallit S (2019). Correlates of self-esteem among the Lebanese population: a cross-sectional study. Psychiatria Danubina.

[58] Mhanna M, Azzi R, Hallit S, Obeid S, Soufia M (2023). Correlates of orthorexia nervosa in a sample of Lebanese adolescents: the co-moderating effect of body dissatisfaction and self-esteem between mental health issues and orthorexia nervosa. Vulnerable Child Youth Stud.

[59] Mundfrom DJ, Shaw DG, Ke TL (2005). Minimum sample size recommendations for conducting factor analyses. Int J Test.

[60] Hu L, Bentler PM (1999). Cutoff criteria for fit indexes in covariance structure analysis: conventional criteria versus new alternatives. Struct Equation Modeling: Multidisciplinary J.

[61] Dash S. M., N. K. (2011). Marketing Research: An Applied Orientation. *Pearson-Dorling Kindersley*, *6th edition, Delhi*.

[62] Chen FF (2007). Sensitivity of goodness of fit indexes to lack of Measurement Invariance. Struct Equation Modeling: Multidisciplinary J.

[63] Vandenberg R, Lance C (2000). A review and synthesis of the Measurement Invariance Literature: suggestions, practices, and recommendations for Organizational Research. Organizational Res Methods.

[64] Fekih-Romdhane F, Malaeb D, Fawaz M, Chammas N, Soufia M, Obeid S, Hallit S (2023). Psychometric properties of an arabic translation of the multidimensional assessment of interoceptive awareness (MAIA-2) questionnaire in a non-clinical sample of Arabic-speaking adults. BMC Psychiatry.

[65] Dunn TJ, Baguley T, Brunsden V (2014). From alpha to omega: a practical solution to the pervasive problem of internal consistency estimation. Br J Psychol (London England: 1953).

[66] Hair J, Sarstedt M, Ringle C, Gudergan S. (2017). *Advanced Issues in Partial Least Squares Structural Equation Modeling*.

[67] Alfonso-Fuertes I, Alvarez-Mon MA, Hoyo RS, del, Ortega MA, Alvarez-Mon M, Molina-Ruiz RM (2023). Time Spent on Instagram and Body Image, Self-esteem, and physical comparison among young adults in Spain: Observational Study. JMIR Formative Res.

[68] Etu SF, Gray JJ (2010). A preliminary investigation of the relationship between induced rumination and state body image dissatisfaction and anxiety. Body Image.

[69] Aggarwal R, Ranjan D, Chandola R (2023). Effect of body image on Self Esteem: a systematic literature review and future implication. Eur Chem Bull.

[70] Nesi J, Prinstein MJ (2015). Using Social Media for Social Comparison and Feedback-Seeking: gender and Popularity Moderate associations with depressive symptoms. J Abnorm Child Psychol.

[71] Wang W, Wang M, Hu Q, Wang P, Lei L, Jiang S. Upward social comparison on mobile social media and depression: the mediating role of envy and the moderating role of marital quality. J Affect Disord. 2020;270. 10.1016/j.jad.2020.03.173. 10.1016/j.jad.2020.03.173 32339106

[72] Schmuck D, Karsay K, Matthes J, Stevic A (2019). Looking up and feeling down. The influence of mobile social networking site use on upward social comparison, self-esteem, and well-being of adult smartphone users. Telematics Inform.

