# Supplementary material for: Psychometric properties of the arabic translation of the Physical Appearance Comparison Scale-Revised (PACS-R) in adults
Source: BMC Psychol. 2024 Jun 29;12:371. doi: 10.1186/s40359-024-01871-x (PMC11218361; doi:10.1186/s40359-024-01871-x)
Supplement: Supplementary file 1 — Supplementary Material 1 [file 40359_2024_1871_MOESM1_ESM.docx]

**Physical Appearance Comparison Scale- Revised- Arabic version**

يقارن الناس أحيانًا مظهرهم الجسدي بمظهر جسد الآخرين. يمكن أن تكون هذه المقارنة بين وزنهم ، حجم الجسم ، وشكل الجسم ، ودهون الجسم أو المظهر العام. بالتفكير في كيفية مقارنة نفسك بالآخرين بشكل عام - يرجى استخدام المقياس التالي لتقييم عدد المرات التي تجري فيها هذه الأنواع من المقارنات.

عندما أكون في الخارج - أقارن مظهري الجسدي بمظهر الآخرين.

عندما أقابل شخصًا جديدًا (من نفس الجنس)، أقارن حجم جسدي بحجم جسمه / جسمها.

عندما أكون في العمل أو المدرسة، أقارن شكل جسدي بشكل جسم الآخرين.

عندما أكون بالخارج في الأماكن العامة - أقارن الدهون في جسدي بدهون أجساد الآخرين.

عندما أتسوق لشراء الملابس، أقارن وزني بوزن الآخرين.

عندما أكون في حفلة، أقارن شكل جسمي بشكل جسم الآخرين.

عندما أكون مع مجموعة من الأصدقاء، أقارن وزني بوزن الآخرين.

عندما أكون في الأماكن العامة، أقارن حجم جسدي بحجم أجساد الآخرين.

عندما أكون مع مجموعة من الأصدقاء، أقارن حجم جسدي بحجم أجساد الآخرين.

عندما آكل في مطعم، أقارن دهون جسدي بدهون الآخرين

عندما أكون في صالة الألعاب الرياضية: أقارن مظهر جسدي بمظهر الآخرين.
